# Hydrogels for Translucent Wearable Electronics: Innovations in Materials, Integration, and Applications

**DOI:** 10.3390/gels11050372

**Published:** 2025-05-20

**Authors:** Thirukumaran Periyasamy, Shakila Parveen Asrafali, Jaewoong Lee

**Affiliations:** Department of Fiber System Engineering, Yeungnam University, Gyeongsan 38541, Republic of Korea

**Keywords:** wearable electronics, hydrogels, physiological sensors, actuators, electronics

## Abstract

Recent advancements in wearable electronics have significantly enhanced human–device interaction, enabling applications such as continuous health monitoring, advanced diagnostics, and augmented reality. While progress in material science has improved the flexibility, softness, and elasticity of these devices for better skin conformity, their optical properties, particularly transparency, remain relatively unexplored. Transparent wearable electronics offer distinct advantages: they allow for non-invasive health monitoring by enabling a clear view of biological systems and improve aesthetics by minimizing the visual presence of electronics on the skin, thereby increasing user acceptance. Hydrogels have emerged as a key material for transparent wearable electronics due to their high water content, excellent biocompatibility, and tunable mechanical and optical properties. Their inherent softness and stretchability allow intimate, stable contact with dynamic biological surfaces. Furthermore, their ability to support ion-based conductivity is advantageous for bioelectronic interfaces and physiological sensors. Current research is focused on advancing hydrogel design to improve transparency, mechanical resilience, conductivity, and adhesion. The core components of transparent wearable systems include physiological sensors, energy storage devices, actuators, and real-time displays. These must collectively balance efficiency, functionality, and long-term durability. Practical applications span continuous health tracking and medical imaging to next-generation interactive displays. Despite progress, challenges such as material durability, scalable manufacturing, and prolonged usability remain. Addressing these limitations will be crucial for the future development of transparent, functional, and user-friendly wearable electronics.

## 1. Introduction

Recent breakthroughs in fields such as biology, electronics, and artificial intelligence have significantly narrowed the boundary between humans and machines. These advancements are leading toward a seamless integration of human and machine systems. This integration not only tackles persistent challenges such as chronic illnesses but also paves the way for enhancing human capabilities in ways previously unimaginable. This growing interest in human–machine fusion has catalyzed the rapid development of multidisciplinary technologies that focus on monitoring, supporting, and improving human health. At present, advanced diagnostic procedures often rely on large, complex machines housed in controlled environments and operated by professionals. While accurate, these systems are limited in their accessibility and are impractical for continuous health monitoring in daily life [1,2,3,4]. This challenge has led to a surge in wearable electronics—compact systems that incorporate miniaturized sensors, energy units, and data processing components. These devices can wirelessly collect and transmit physiological signals from the human body, allowing for real-time health tracking even in remote settings. Popular consumer products like the Apple Watch exemplify this shift toward decentralized healthcare, where individuals can monitor aspects of their health outside traditional clinical environments. As a result, the entire medical paradigm is evolving from reactive treatment in fixed locations to proactive monitoring and intervention in real time, regardless of location.

This transition toward “space-free” healthcare has begun to influence other industries as well, such as remote communication, virtual commerce, and online job training. For instance, technologies like the metaverse rely heavily on this integration, signaling the broader implications of wearable systems in our digital lives. However, most current wearable devices, including smartwatches, fitness bands, and headsets for extended reality (XR), rely on rigid materials such as metal and silicon. These materials, while durable and electronically efficient, do not conform well to the human body’s soft, curvilinear surfaces. This mismatch in mechanical properties results in poor contact with the skin, decreased signal quality, and discomfort during long-term use. Consequently, many users may be discouraged from wearing such devices regularly [3,4,5,6].

Recent advances in hydrogel-based materials have transformed their potential for wearable electronics, owing to their tunable mechanical strength, ionic/electronic conductivity, and multifunctional responsiveness. Liu et al. developed electrically conductive hydrogel-based microelectronics with tissue-like Young’s modulus and exceptional electrochemical stability, enabling localized, low-voltage neuromodulation through a micropatterned, biocompatible structure that addresses chronic implantation challenges [7]. Complementing this, Tordi et al. introduced multiresponsive alginate-gelatin organohydrogels capable of responding to temperature, humidity, strain, and light due to ionic cross-linking with multivalent cations, offering high transparency and biocompatibility for real-time biosensing and wearable e-skin applications [8].

Meanwhile, Li et al. engineered a hierarchical PEDOT:PSS/PVA organohydrogel with outstanding mechanical robustness (tensile strength up to 54.8 MPa, toughness of 153.97 MJ/m^3^) and ultra-sensitive strain sensing (gauge factor of 983), enabled by semi-interpenetrating networks and crystallinity-enhancing fabrication methods [9]. Collectively, these innovations showcase the convergence of biocompatibility, structural integrity, and multifunctional sensing in hydrogel design, which are key to advancing next-generation wearable electronics for health monitoring, human–machine interfaces, and environmental sensing.

**Scheme 1:**
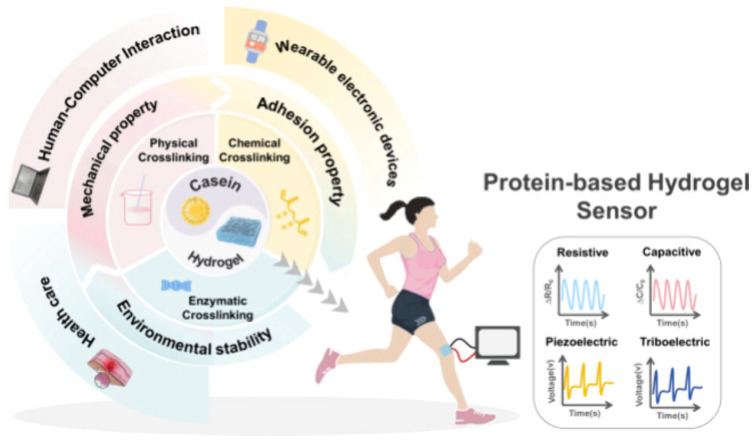
Preparation techniques, performance enhancement strategies, sensing mechanisms, and diverse applications of protein-based hydrogel sensors from the standpoint of casein [17].

Hydrogels are three-dimensional (3D) polymeric networks that can absorb and retain large quantities of water while maintaining their structure due to chemical or physical cross-linking. This unique combination of softness, high water content, and tunable mechanical properties makes hydrogels highly attractive for applications in wearable electronics, where biocompatibility, flexibility, and adaptability are essential. In the context of wearable electronics, hydrogels must meet several demanding criteria [10,11,12,13]. These include robust mechanical integrity to withstand repeated deformation and stretching, strong adhesion to various substrates (including skin), long-term environmental stability, and high conductivity for effective signal transduction. As wearable devices are often used in dynamic and moist environments, hydrogels must also exhibit excellent stretchability, fatigue resistance, and resilience under mechanical strain. The mechanical properties critical to hydrogel performance in wearable applications include tensile strength, elasticity, compressibility, and toughness. These characteristics enable the hydrogel to conform to complex body surfaces without cracking or delaminating. On the other hand, the key electronic properties involve high ionic or electronic conductivity, low impedance, and stable performance over time. These properties are vital for accurate signal transmission in sensors and for functioning as electrodes or interconnects in bioelectronic systems. Advanced hydrogel formulations often integrate conductive fillers such as carbon nanotubes, metallic nanoparticles, or ionic salts to enhance electronic performance without compromising their mechanical flexibility [12,13,14,15,16,17]. The ideal hydrogel for wearable electronics strikes a balance between mechanical robustness and electronic functionality, enabling its integration into soft, skin-like devices that can monitor physiological signals, deliver therapeutic stimuli, or support interactive interfaces (Scheme 1).

Integrating transparency into wearable devices would preserve visual access to the underlying biological tissue. This transparency can be utilized in combination with biosensor data to offer a more comprehensive understanding of a user’s health. Furthermore, when paired with vision-based sensors and artificial intelligence, transparent electronics can enable advanced data processing, perception, and prediction. This integration opens new avenues for precision control in robotics and wearable actuators, particularly those responding to human movement.

In XR environments, transparent wearable devices can also enhance user experience by aligning the visual representation of the body with sensory feedback, minimizing dissonance between what the user sees and feels. This synchronization boosts cognitive processing and immersion, enabling a more natural interaction between the virtual and physical worlds. The result is a more seamless, immersive, and realistic XR experience. From a futuristic standpoint, the concept of “optical imperceptibility”, in which electronic devices become visually unnoticeable, plays a pivotal role. This transparency reduces psychological resistance to wearing the devices daily and helps merge virtual and real environments more effectively. When combined with mechanical imperceptibility, it offers a transformative approach to the design of wearable electronics [16,17,18,19]. These qualities address current limitations such as discomfort, visual obstruction, and disjointed user experiences between physical and digital domains.

In this context, recent research has delved into developing transparent materials for wearable applications. These materials are classified into categories such as electrodes, active layers, and encapsulation components. Each category comes with unique properties and synthesis methods tailored to achieve high transparency while maintaining excellent electronic performance. Additionally, engineering approaches are being employed to optimize the balance between optical clarity and electrical functionality, particularly in electrodes and active components. Strategies to ensure mechanical imperceptibility, such as improved stretchability, softness, and stability over long-term use, are also evolving. This includes modifying conventional rigid structures and adopting intrinsically flexible materials. Furthermore, this progress extends to the entire ecosystem of transparent wearable devices, encompassing sensors, power sources, and actuators. Each component is being engineered for optimal performance while contributing to the overall transparency and comfort of the device.

Looking ahead, wearable electronics with both optical and mechanical imperceptibility hold tremendous potential. From healthcare and virtual reality to robotics and smart environments, these next-generation devices have the potential to transform the way we engage with our bodies and the surrounding world. Biopolymers like gelatin, cellulose, lignin, and chitosan are promising natural hydrogel substrates due to their biocompatibility and eco-friendliness, making them safer alternatives to synthetic materials. Their high water content and porous structure support wound healing, but limitations such as low gauge factor (GF) and poor mechanical durability, especially under joint movement, could hinder their broader use. These weaknesses can lead to wound re-tearing and infection. To address this, recent efforts focus on multifunctional conductive hydrogel dressings with self-healing, injectability, bacterial theranostics, and motion sensing. Enhancing GF and cyclic stability is key to realizing durable, responsive, next-generation wound care materials [20,21,22,23,24]. However, challenges remain, including improving long-term durability, data security, and user adaptability—areas that future research will continue to address.

### 1.1. Advanced Fabrication of Elastomeric Ionic Hydrogel for Moisture-Electric Generation in Wearables

As the demand for sustainable, flexible, and portable energy sources continues to rise, especially in the realm of wearable electronics, innovative strategies for energy harvesting are gaining increased attention. Among these, moisture-electric energy conversion stands out as a promising solution for powering next-generation wearable devices without reliance on traditional batteries. In this context, Guchait et al. [25] introduced a novel approach to harvesting ambient moisture and converting it into usable electrical energy through a flexible and stretchable moisture-electric generator (FMEG). This device is based on an ionic hydrogel platform and addresses many of the limitations found in conventional energy harvesting systems, such as mechanical rigidity, limited energy output, and environmental unfriendliness. The foundation of the FMEG lies in its specially engineered hybrid hydrogel, which integrates polyurethane (PU), polyacrylamide (PAAm), and calcium chloride (CaCl_2_). This unique composition allows for both high mechanical elasticity and efficient ionic conductivity.

**Figure 1:**
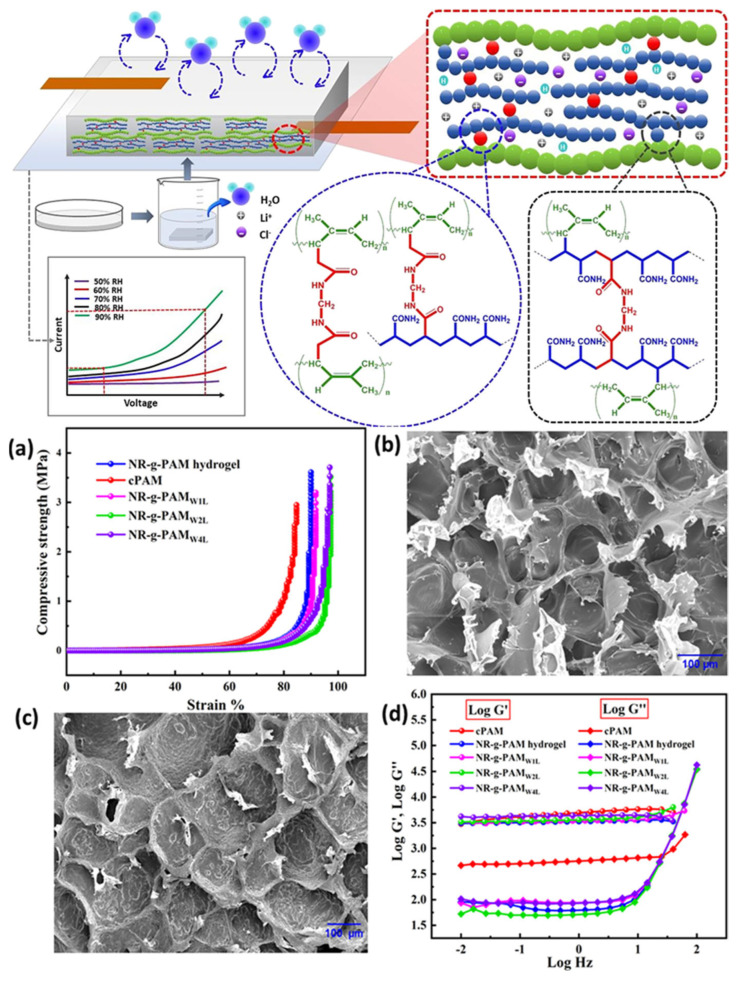
The Chemical Structure of the Ionic Hydrogel, MEG Preparation Process. (**a**) Compressive strength comparison among the NR-g-PAM hydrogel. (**b**) SEM image of the freeze-dried cPAM hydrogel. (**c**) SEM image of the freeze-dried NR-g-PAM hydrogel. (**d**) Logarithmic storage modulus (log G′) and loss modulus (log G″) versus logarithmic frequency [25].

The preparation process involved the in-situ polymerization of acrylamide monomers within a PU network, followed by a solvent exchange method that incorporated hygroscopic calcium chloride. This integration was key to ensuring the hydrogel’s ability to absorb moisture from the surrounding air efficiently. Characterization techniques such as Fourier-transform infrared spectroscopy (FTIR) and scanning electron microscopy (SEM) were employed to verify the chemical bonding between the components and the uniform distribution of ions throughout the polymer matrix. The resulting hydrogel demonstrated several desirable properties: mechanical robustness, transparency, adhesiveness, and high ionic mobility—critical features for energy harvesting in flexible applications, as shown in Figure 1 [26,27,28].

The energy harvesting capability of the FMEG is grounded in its ability to generate electrical voltage through moisture-induced ion migration. Calcium chloride, a highly hygroscopic salt, plays a central role in this process by absorbing moisture from the environment. This leads to an increase in the water content of the hydrogel, which can reach up to 74.6% by weight at a relative humidity (RH) of 90%. The absorbed moisture promotes the dissociation of calcium and chloride ions within the matrix. A naturally occurring vertical gradient of moisture, where the upper surface of the film is more hydrated than the bottom, creates a directional pathway for ion migration. This directional ion flow generates a potential difference across the thickness of the hydrogel, which can be harnessed as electrical output. Under controlled conditions (80% RH), the FMEG was able to produce an open-circuit voltage (Voc) of 0.98 volts and a short-circuit current density of 0.38 μA/cm^2^, which ranks among the highest reported values for hydrogel-based moisture harvesters. Moreover, the electrical performance scaled positively with increasing humidity levels, and the voltage remained stable over prolonged operation. A significant highlight of the device is its mechanical durability. Thanks to the elastomeric nature of the PU matrix, the FMEG retained its electrical output even under physical deformations such as stretching (up to 50% strain), twisting, and bending. This level of mechanical resilience is particularly important for wearable devices, which are subject to constant movement and dynamic strain during use [29,30].

To assess the practicality of this hydrogel-based energy harvesting platform, Guchait et al. adapted the FMEG into wearable formats. The hydrogel’s inherent adhesiveness allowed for direct attachment to human skin and integration with textiles without the need for additional adhesives or mechanical supports. A compelling demonstration involved powering a digital wristwatch solely through the ambient moisture present in the air and skin perspiration. When worn on the wrist, the device continuously generates electricity, eliminating the need for external power sources or batteries. This real-time energy harvesting showcased the viability of the FMEG for powering low-energy wearable electronics in typical environmental conditions.

**Figure 2:**
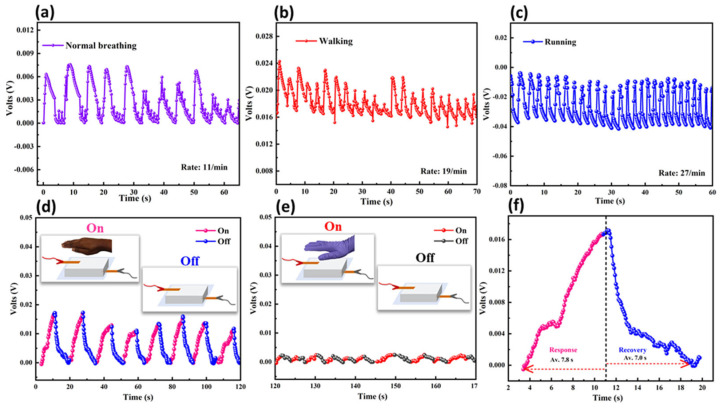
Illustration of the self-powered sensor integrated into a mask for monitoring breathing patterns during (**a**) normal sitting, (**b**) walking, and (**c**) running. Detection of hand proximity is shown for (**d**) a bare hand and (**e**) a gloved hand. (**f**) Response and recovery times of the sensor during hand proximity detection [25].

Furthermore, dynamic motion testing was conducted by affixing the device to different parts of the body, including joints such as the elbow and wrist. Even during repeated bending, folding, and stretching motions, the FMEG maintained consistent voltage output, confirming its robustness in real-world scenarios. The adhesive hydrogel not only provided a secure, conformal interface with the skin but also enhanced user comfort, essential for applications like epidermal sensors, sweat-based monitors, and skin-compatible energy harvesters. One of the major advantages of this energy harvesting technology lies in its sustainability. The hydrogel components—PU, PAAm, and CaCl_2_—are biocompatible, non-toxic, and biodegradable, making the device safe for prolonged contact with human skin and environmentally benign. Additionally, the fabrication process is eco-conscious, involving minimal solvent usage and avoiding harsh chemicals, thus aligning with broader goals of green technology development. Importantly, the device demonstrated reusability across multiple operational cycles. The electrical performance remained above 90% of its initial output even after 100 cycles of mechanical deformation, as illustrated in Figure 2a–f. Similarly, the hydrogel maintained function across numerous moisture absorption–desorption cycles, confirming its long-term stability and effectiveness.

In summary, the flexible moisture-electric generator developed by Guchait et al. represents a significant advancement in the field of self-powered wearable electronics. By leveraging the synergy between materials science and sustainable engineering, this hydrogel-based platform offers a robust, environmentally friendly, and highly adaptable solution for ambient energy harvesting. Its ability to operate reliably under variable humidity and mechanical stress positions it as a strong candidate for incorporation into future wearable devices, paving the way for a new generation of battery-free, self-sustaining electronic systems.

### 1.2. Versatile and Simple Strategy for Preparing Bilayer Hydrogels with Janus Characteristics

**Figure 3:**
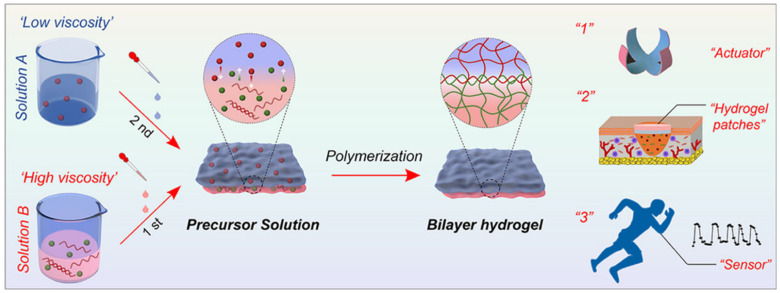
Schematic representation of the one-step fabrication strategy for bilayer hydrogels [31].

As the need for multifunctional soft materials rises across fields like soft robotics, bioengineering, and wearable technology, He et al. [31] proposed a simple yet effective approach for engineering bilayer hydrogels with Janus characteristics—materials that exhibit distinct properties on opposing sides. These hydrogels are designed to meet increasing demands for programmable, responsive materials that mimic natural systems and adapt to various stimuli such as humidity, temperature, and mechanical stress. The team developed a generalizable, facile strategy to construct bilayer hydrogels via dynamic interfacial bonding, offering a powerful new platform for smart materials and devices. The innovative fabrication process leverages dynamic Schiff-base bonds and hydrogen bonding at the interface of two chemically distinct hydrogel layers, as shown in Figure 3. The top layer typically consists of a poly(vinyl alcohol) (PVA)-based hydrogel that incorporates aldehyde-containing groups, while the bottom layer is composed of a gelatin-based hydrogel, rich in amino functionalities. When these layers are brought into contact, interfacial cross-linking occurs spontaneously under ambient conditions without the need for harsh reagents, UV light, or initiators. This approach allows for rapid, scalable fabrication of bilayer hydrogels with controllable properties across both faces [32,33,34].

A major advantage of this method lies in its adaptability. By varying the composition and structure of the individual layers, a broad library of bilayer combinations can be created to meet different application requirements. The researchers utilized reversible Schiff-base bonding (between aldehyde and amine groups) alongside hydrogen bonding to achieve robust yet dynamic interfaces. Fourier-transform infrared spectroscopy (FTIR) and scanning electron microscopy (SEM) confirmed the successful formation of a unified bilayer interface. The interface appeared seamless and highly integrated, enabling effective stress transfer and maintaining mechanical coherence during actuation or deformation. The adhesion strength between the layers was significantly enhanced by the dual bonding strategy. Unlike conventional hydrogels, where delamination is a common issue under cyclic mechanical stress, these Janus bilayers exhibited excellent interfacial toughness and could endure bending, stretching, and repeated cycles of actuation without structural failure. The bonding mechanism is also reversible, allowing for repairability and reusability—key characteristics for sustainable material systems [31,34,35].

In terms of mechanical performance, the bilayer hydrogels demonstrated high stretchability, flexibility, and toughness. The PVA-rich layer contributed to mechanical strength and water retention, while the gelatin-based layer provided softness and biocompatibility. The mechanical properties could be fine-tuned by adjusting polymer concentration, cross-linking time, and functional group density. Tensile tests revealed elongation at break values exceeding 400% in some samples, with Young’s moduli tailored based on intended applications. Anisotropic swelling—where one layer swells more than the other upon water absorption—was a key property enabling directional actuation. This behavior is fundamental to the hydrogel’s ability to bend or curl predictably in response to external stimuli. When exposed to humidity or temperature gradients, the swelling mismatch between layers induced mechanical deformation, converting chemical or environmental energy into mechanical motion. These controlled deformations are essential for developing smart actuators or bioinspired soft devices [34,35,36].

**Figure 4:**
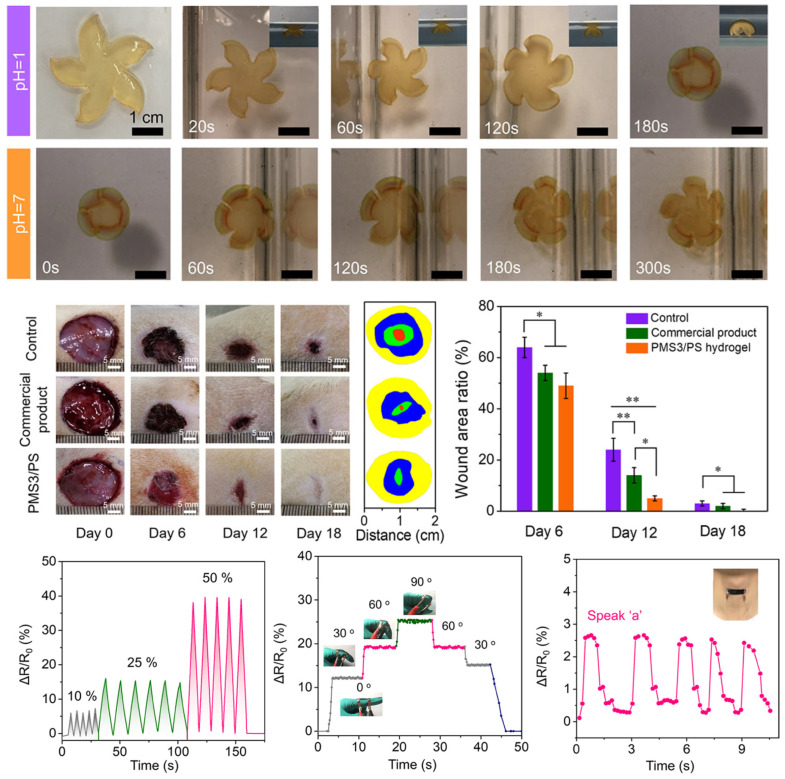
Optical microscopy images showing pH-triggered deformation of the PAAS3/PAA@Fe^3^⁺ gel flower (scale bar: 1 cm). Wound images and corresponding wound area ratios for the blank group (no treatment), reference group (commercial product), and PMS3/PS gel-treated group over time (scale bars: 5 mm) [31].

One of the most impactful aspects of this study is the demonstration of programmable, stimuli-responsive behavior. By designing the geometry, thickness ratio, and composition of each hydrogel layer, the researchers were able to guide the shape transformation in a highly controllable manner. For example, rectangular strips of bilayer hydrogel curled into tubes, spirals, or cones depending on the stimuli and structure. These transformations occurred rapidly (within seconds) and were reversible over multiple cycles, as indicated in Figure 4. In environmental responsiveness tests, the bilayer hydrogels showed significant actuation when subjected to relative humidity changes, making them ideal for humidity sensors or moisture-powered actuators. Similarly, when exposed to temperature fluctuations, thermally induced bending or folding was observed. This dual responsiveness expands the hydrogels’ application potential across fields, such as wearable sensing, robotics, and medical diagnostics.

To validate the practical applications of their material system, He et al. integrated the bilayer hydrogels into various proof-of-concept devices. These included self-curling soft grippers capable of lifting small objects, biomimetic flowers that opened and closed with humidity changes, and thermally triggered bending sensors. The actuators demonstrated stable and repeatable performance under real-world conditions, retaining function over dozens of actuation cycles. The team also highlighted potential applications in wearable electronics, leveraging the biocompatibility and flexibility of the hydrogels. Because gelatin is a widely accepted material in biomedical devices, and the aldehyde-modified PVA is non-toxic, these Janus hydrogels can be safely applied to skin or incorporated into implantable systems. The adhesive nature of the hydrogels also allows direct application onto human skin without needing auxiliary adhesives or tapes, enhancing comfort and usability in wearable systems [31,32,33,34,35,36].

The authors placed strong emphasis on sustainability and environmental safety. The materials used—gelatin and modified PVA—are biodegradable and non-toxic, making the hydrogels safe for biological and environmental interactions. Moreover, the fabrication process is solvent-free and energy-efficient, relying on ambient conditions and avoiding harmful catalysts or initiators. This aligns well with growing global priorities around green chemistry and sustainable materials design. Durability tests showed that the hydrogels retained over 85% of their actuation performance after 100 humidity cycles, and more than 90% structural integrity after multiple swelling–deswelling processes. The use of dynamic bonds at the interface also enabled self-healing capabilities and extended material lifespan, reducing the need for frequent replacement. He et al.’s work introduces a novel yet simple method for constructing bilayer hydrogels with Janus properties, offering exceptional versatility, mechanical robustness, and environmental adaptability. The dynamic bonding interface provides durable interlayer adhesion, while the anisotropic swelling behavior imparts stimuli-responsiveness for smart actuation. These bilayer hydrogels not only demonstrate high potential in soft robotics and wearable electronics but also serve as an environmentally conscious material platform for the next generation of multifunctional soft systems.

### 1.3. Polyelectrolyte Hydrogel with Piezoelectricity, Adhesion, and Self-Healing for Soft Electronics

**Figure 5:**
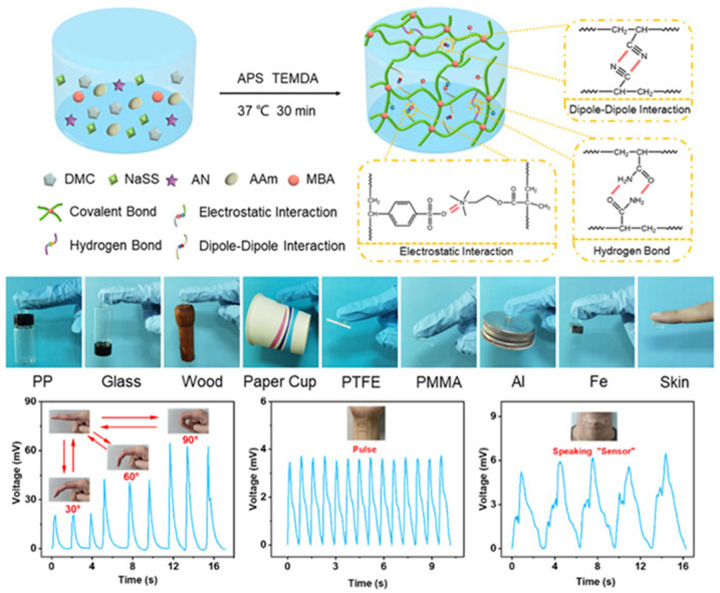
Schematic illustration of the preparation process for PN_x_D_y_A_z_ hydrogels. Evaluation of the adhesive performance of PN_x_D_y_A_2_ hydrogels. Demonstration of the PN_1.04_D_0.96_A_2_ hydrogel applied as a sensor [37].

As the field of soft electronics continues to expand, driven by applications in wearable sensors, bio-integrated devices, and human–machine interfaces, the need for multifunctional and durable materials becomes increasingly apparent. Liu et al. (2024) [37] successfully synthesized a polyelectrolyte hydrogel (PN_x_D_y_A_z_) via copolymerization of acrylonitrile (AN), acrylamide (AAm), methacryloxyethyltrimethylammonium chloride (DMC), and sodium p-styrenesulfonate (NaSS) (Figure 5). The hydrogel formation was driven by electrostatic interactions between the oppositely charged polyelectrolytes DMC and NaSS, embedded within a polyacrylonitrile (PAN)-based piezoelectric matrix. Incorporation of the piezoelectric monomers AN, DMC, and NaSS into the hydrogel network was confirmed, yielding a material with excellent mechanical and functional properties. The PN_1.04_D_0.96_A_2_ hydrogel exhibited outstanding mechanical strength, achieving a fracture stress of 140.65 ± 4.52 kPa and a fracture strain of 499 ± 9.54%. These properties were attributed to a synergistic network structure reinforced by dipole–dipole interactions of the cyano groups and intermolecular hydrogen bonding, which together enhanced cross-linking density and facilitated efficient energy dissipation [38,39]. The inclusion of AN further contributed to a gradual increase in tensile strength and Young’s modulus. In terms of mechanical durability, the hydrogel demonstrated remarkable fatigue resistance under cyclic deformation, as evidenced by stable performance over 50 loading–unloading cycles.

**Figure 6:**
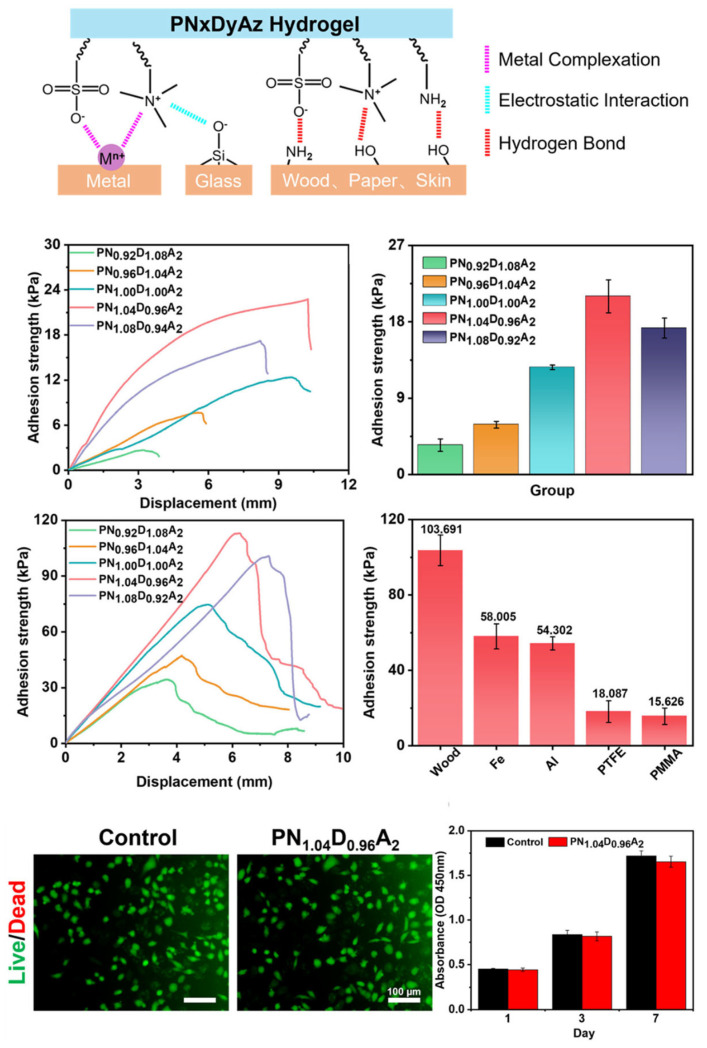
Adhesive and biocompatibility performance of PN_x_D_y_A_2_ hydrogels, adhesion, and strength on pigskin. Lap shear test on wood. Lap shear strength on various substrates. Live/dead staining of fibroblasts (L929) after 3 days with or without PN_1.04_D_0.96_A_2_ hydrogel extract. CCK-8 assay showing fibroblast proliferation [37].

In addition to mechanical robustness, the PN_x_D_y_A_z_ hydrogel showed strong adhesion to biological substrates, with an adhesion strength of 22.78 kPa on pig skin (Figure 6). This adhesive capability arises from the presence of both positive and negative groups, introduced by DMC and NaSS, which form noncovalent interactions such as metal ion coordination, electrostatic attraction, and hydrogen bonding with various surfaces [40]. The hydrogel also demonstrated excellent piezoelectric performance. Under external pressure, the strong dipole moment of the cyano groups in PAN led to the reorientation and alignment of free cyano groups, generating a potential difference across the hydrogel. With increasing AN content, the output voltage was significantly enhanced from 8.00 ± 0.29 mV to 70.33 ± 0.79 mV [41]. Functionally, the PN_1.04_D_0.96_A_2_ hydrogel was evaluated as a wearable strain sensor. It accurately captured finger bending at 0°, 30°, 60°, and 90° (Figure 5), as well as joint movements of the elbow and knee. Beyond large-scale motion detection, the hydrogel was sensitive to subtle physiological signals. When attached to the wrist, it recorded a volunteer’s pulse, and when positioned on the throat, it detected vocal cord vibrations during speech, such as the articulation of the word “sensor” [42]. These results highlight the hydrogel’s potential for applications in voice recognition and real-time health monitoring.

Biocompatibility was confirmed through both live/dead staining and CCK-8 assays. Minimal red fluorescence indicated low levels of dead cells in both the hydrogel and control groups, and cell viability was comparable between them, as shown in Figure 6. These findings confirm that the PN_1.04_D_0.96_A_2_ hydrogel does not induce cytotoxicity, skin irritation, or allergic responses, even during prolonged use.

### 1.4. Fabrication of High-Toughness, Puncture-Resistant Hydrogels Based on Nanoengineered MXene for Flexible Wearable Electronics

**Figure 7:**
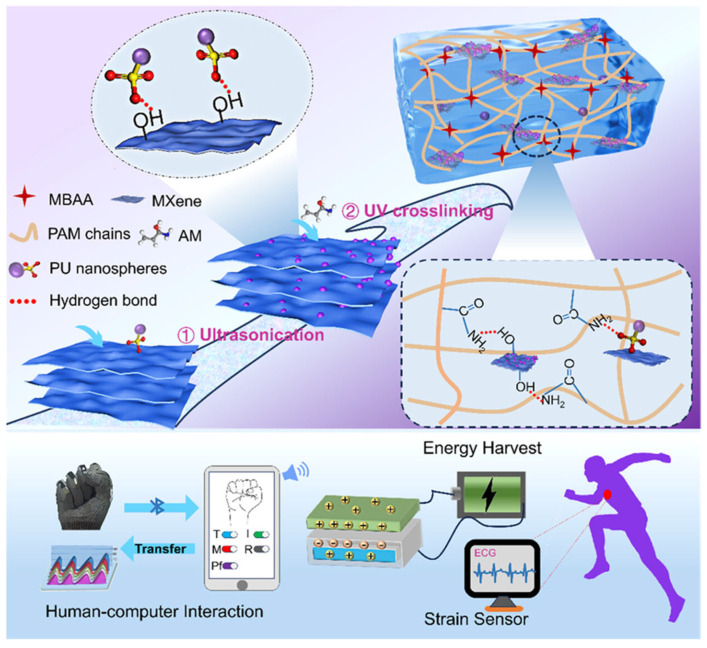
Schematic and fabrication process of the PUMP hydrogels and further applications [43].

As wearable electronics continue to evolve toward more dynamic, flexible, and durable systems, materials that combine mechanical resilience, flexibility, and electrical conductivity have become crucial. In response to these demands, Lin et al. (2024) [43] developed a highly tough, ultrathin, and puncture-resistant hydrogel using a nanoengineering strategy centered on strong hydrogen bonding interactions between sulfonate-modified polyurethane (PU) nanospheres and MXene nanosheets (Figure 7). In this design, the PU nanospheres acted as effective dispersing agents, significantly improving the uniform distribution of MXene while simultaneously enhancing the flexibility and stretchability of the hydrogel. Specifically, the hydroxyl-rich surface of MXene formed robust hydrogen bonds with sulfonic acid groups on the PU nanospheres, effectively weakening the interlayer Coulombic forces between MXene sheets and promoting their stable dispersion within the hydrogel matrix [44,45]. To maintain this dispersion and inhibit MXene restacking, a cross-linked polyacrylamide (PAM) network was introduced, enabling the fabrication of an ultrathin hydrogel film with a thickness of just 32 μm. The mechanical performance of the resulting hydrogel was exceptional. Owing to the inherent elasticity of PU, the PUMP hydrogel (PU@MXene/PAM Hydrogel) exhibited outstanding toughness (385.6 kJ/m^3^) and high stretchability, sustaining elongation up to 10 times its original length without failure. As the concentration of PU nanospheres increased, stretchability improved significantly, reaching a maximum of 1834%. Additionally, the tensile strength of the hydrogel increased with PU content, which can be attributed to the strong hydrogen bonding interactions between sulfonate-functionalized MXene and the PAM network [46].

The ultrathin PUMP hydrogel film also demonstrated excellent surface conformability and optical clarity. When adhered to a volunteer’s palm, it conformed seamlessly to the skin, allowing fine palmprint details to be observed with the naked eye. Furthermore, the hydrogel adhered effectively to various substrates, including foam, glass, wood, and metal. Among samples of varying thicknesses, the 1 mm-thick hydrogel displayed the best puncture resistance. In contrast, thinner hydrogel films exhibited lower stiffness and greater deformability, resulting in enhanced displacement under lower applied forces. This geometric flexibility is a critical advantage for ultrathin hydrogel-based materials [47]. In addition to its mechanical robustness, the PUMP hydrogel also showed excellent sensitivity as a strain sensor. It exhibited a gauge factor of 2.14 within the 0–200% strain range and 4.5 within 200–700%, indicating a substantial resistance change under deformation due to the dynamic reconfiguration of the conductive network [48]. When applied to a volunteer’s wrist and subjected to cyclic bending, the hydrogel sensor demonstrated a fast response time of 0.18 s and a recovery time of 0.20 s, confirming its responsiveness and stability for real-time strain monitoring.

**Figure 8:**
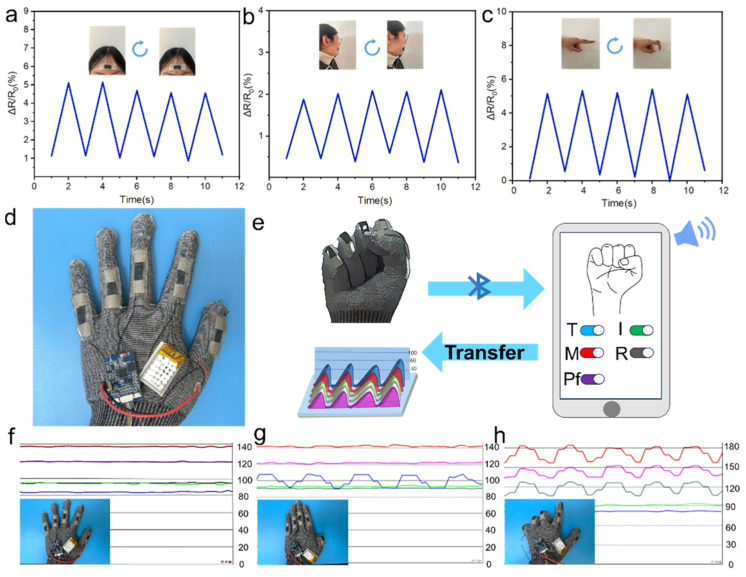
Relative resistance changes of PUMP hydrogel for monitoring human motions in real-time. Bending and recovery process of (**a**) frowning, (**b**) gurgling, and (**c**) the finger. (**d**) Illustration of the smart glove. (**e**) The schematic diagram of the “smart glove” sensing system. The real-time recording of (**f**) open hand, (**g**) one finger, and (**h**) three fingers [43].

The versatility of the hydrogel sensor was further validated through a range of human motion sensing applications. As shown in Figure 8, it was attached to a volunteer’s forehead, where it successfully detected subtle facial expressions, such as frowning, by measuring changes in electrical resistance induced by sensor deformation. It also captured changes in signal output when the volunteer’s cheek protruded or relaxed, showcasing its ability to detect fine facial muscle movements. Beyond subtle expressions, the sensor reliably monitored large-scale motions such as finger bending and straightening, with clear variations in relative resistance, enabling accurate real-time tracking of joint movement. These results collectively highlight the PUMP hydrogel sensor’s exceptional sensitivity, flexibility, and multifunctionality, making it a promising candidate for next-generation wearable electronics and human–machine interface technologies.

### 1.5. Photoinitiated Synthesis of Electrically Conductive Hydrogels for Bioelectronics Applications

As wearable and implantable bioelectronic devices drive advances in health monitoring and soft robotics, there is a growing demand for materials that effortlessly integrate mechanical flexibility, electrical conductivity, and biocompatibility. In response to this, Nguyen et al. (2023) [49] proposed a groundbreaking and simplified one-pot, photomediated approach to fabricate electrically conductive hydrogels (ECHs). These hydrogels offer a unique combination of stretchability, adhesion, and conductivity, making them ideal for skin-compatible, soft electronics. Traditional fabrication methods of conductive hydrogels typically involve complex, multi-step procedures that often compromise between conductivity, mechanical robustness, and environmental or biological compatibility. Nguyen et al. sought to address these limitations by developing a facile and scalable one-step UV-initiated polymerization method, resulting in ECHs that retain excellent physical properties without the drawbacks of conventional processes.

**Figure 9:**
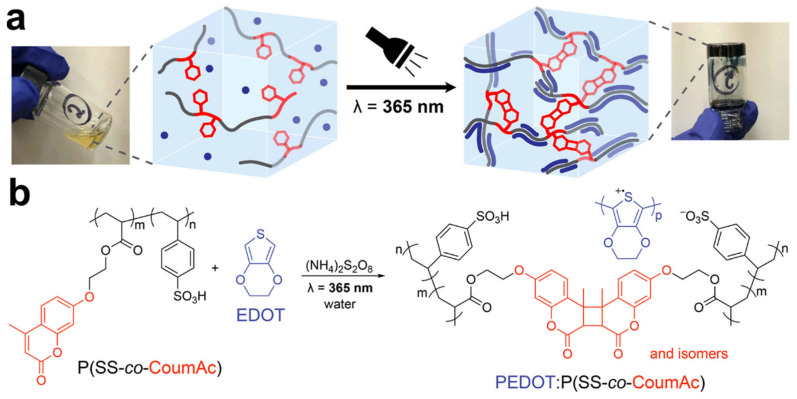
Photomediated Formation of Conductive Hydrogels. (**a**) Images and schematics showing the precursor solution (left) and the conductive hydrogel formed after light exposure (right). (**b**) Simultaneous light-induced cross-linking of P(SS-co-CoumAc) via [2 + 2] cycloaddition and oxidative polymerization of EDOT’s mechanical and electrical performance [49].

The fabrication process involved a photo-initiated copolymerization of zwitterionic monomers and a conductive polymer dispersion in an aqueous environment. Specifically, the hydrogel matrix was synthesized using poly(3,4-ethylenedioxythiophene):polystyrene sulfonate (PEDOT:PSS) as the conductive component, and [2-(methacryloyloxy)ethyl]dimethyl-(3-sulfopropyl)ammonium hydroxide (SBMA) as the zwitterionic monomer, combined with a cross-linker (MBAA) and photoinitiator (LAP). Under UV exposure, these ingredients formed an interpenetrating polymer network that encapsulated PEDOT:PSS uniformly throughout the gel, as illustrated in Figure 9. This one-pot synthesis route stands out for its simplicity, speed (completed in minutes), and versatility. PEDOT:PSS forms nanoscale conductive pathways, while the zwitterionic matrix promotes mechanical toughness, water retention, and antifouling properties [49,50,51,52]. Notably, SEM imaging showed a highly porous structure that is ideal for both ion and electron transport. FTIR spectroscopy further confirmed the strong hydrogen bonding and electrostatic interactions between PEDOT:PSS and the SBMA-based network, enhancing both the mechanical integrity and electrical coupling.

A critical achievement of this work is the balance between high electrical conductivity (~7.1 S/m) and mechanical stretchability (>600% strain). Many conductive hydrogels suffer a trade-off: higher conductivity often means brittleness or decreased stretchability. However, Nguyen et al. demonstrated that by tuning the ratio of PEDOT:PSS and SBMA, they could achieve high conductivity while preserving elasticity and softness. The ECHs with optimized composition maintained their conductivity even under high strain, bending, and cyclic stretching. In fatigue tests, the hydrogels were subjected to repeated elongation (over 1000 cycles), during which the electrical resistance remained relatively stable. This mechanical-electrical robustness is essential for wearable applications that require materials to withstand repeated deformation. Additionally, the self-adhesive properties of the ECHs allowed them to adhere strongly to skin and other surfaces without requiring external adhesives. This adhesion is attributed to the zwitterionic components, which facilitate strong interactions with both dry and wet surfaces, including human skin, metal, glass, and plastic [52,53,54].

**Figure 10:**
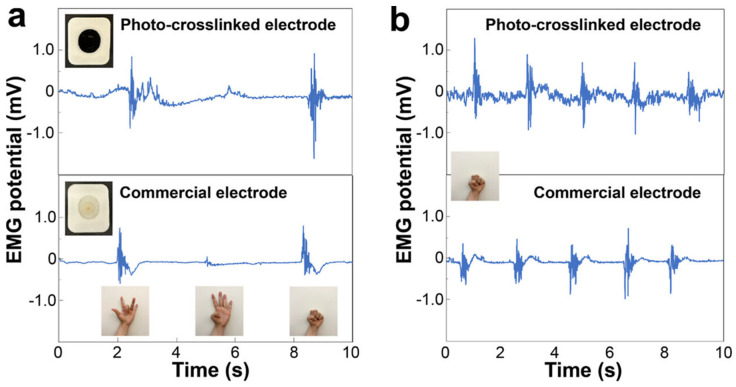
Comparison of sEMG signals from a photo-cross-linked electrode (top) and a commercial 3M electrode (bottom) on the forearm: (**a**) during various finger movements and (**b**) during repeated fist closures. The insets show electrode images and corresponding hand motions [49].

To validate real-world applicability, Nguyen et al. integrated the developed ECHs into bioelectronic interfaces specifically as electrodes for surface electromyography (EMG) signal acquisition. These hydrogel-based electrodes were tested against conventional Ag/AgCl electrodes and demonstrated comparable, if not superior, performance in terms of signal clarity, stability, and resistance to motion artifacts. EMG recordings from forearm muscle movements showed that the hydrogel electrodes produced clear, repeatable signals with minimal noise, even during stretching or motion (Figure 10). Their low skin–electrode impedance ensured high signal fidelity, while their conformal contact minimized discomfort during long-term wear. The hydrogels also demonstrated the potential for reusability; after removal and reapplication, signal performance remained consistent. Their integration into on-skin wearable electronics paves the way for a wide range of applications—from health monitoring and rehabilitation therapy to prosthetic control and human–machine interfaces. Moreover, the compatibility with 3D printing techniques suggests scalability for manufacturing custom-shaped devices suited to individual users or application-specific geometries.

A significant highlight of this work is the biocompatibility and environmental safety of the materials and fabrication method. Cytotoxicity assays confirmed that the hydrogels were non-toxic and safe for prolonged skin contact. The zwitterionic component (SBMA) also imparts antifouling properties, reducing the risk of biofilm formation and ensuring stable long-term performance in biological environments. The ECHs retained both electrical and mechanical functionality after being stored in aqueous environments for over 30 days, and they withstood over 50 cycles of deformation and adhesion/removal without degradation in performance. This reusability and durability, combined with a solvent-free, eco-friendly fabrication process, make these hydrogels a promising candidate for sustainable wearable bioelectronics. Nguyen et al. also highlighted that the synthesis process does not rely on toxic solvents or high temperatures, which enhances its potential for environmentally responsible production. The materials themselves can be processed under ambient conditions using low-energy UV light, further emphasizing their green credentials.

The study by Nguyen et al. (2023) [49] presents a major advancement in the development of multifunctional conductive hydrogels through a simple, scalable, and environmentally friendly one-pot photomediated process. By integrating high stretchability, conductivity, adhesion, and biocompatibility, these ECHs overcome the limitations of traditional hydrogel fabrication methods. Their successful demonstration in EMG acquisition and epidermal electronics reveals broad potential for next-generation wearable devices, particularly in healthcare monitoring, biomedical sensing, and flexible electronic interfaces. Moreover, the versatility of the photopolymerization method, compatibility with additive manufacturing, and robust environmental performance offer exciting prospects for further customization and commercial scalability. In summary, this study presents a robust materials platform that successfully integrates high functional performance with manufacturing simplicity, marking a significant step forward in the development of soft, intelligent, and sustainable bioelectronic materials.

### 1.6. Self-Healable Conductive Hydrogels with High Stretchability and Ultralow Hysteresis for Soft and Wearable Strain Sensors

**Figure 11:**
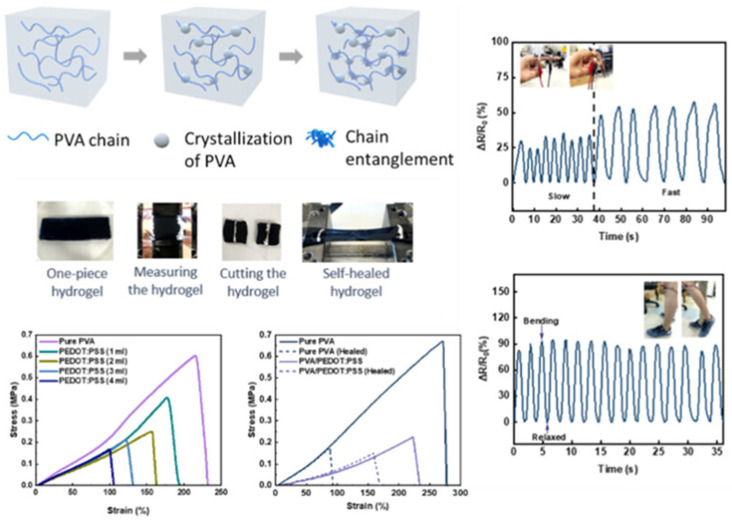
Schematic of the preparation process and properties of the PVA/PEDOT:PSS hydrogel [55].

As the demand for durable, stretchable, and skin-compatible sensors increases in the field of soft electronics and wearable devices, Prameswati et al. [55] proposed a new class of high-performance hydrogels based on poly(vinyl alcohol) (PVA) and poly(3,4-ethylenedioxythiophene):poly(styrenesulfonate) (PEDOT:PSS) for use in soft electronics. The research addresses critical limitations in current hydrogel-based stretchable electronics, particularly issues with low sensitivity, long response time, poor mechanical recovery, and high electrical hysteresis. The authors developed a fabrication method that integrates PEDOT:PSS into a PVA matrix, followed by post-treatment with sodium chloride (NaCl). This strategy significantly enhanced both the mechanical robustness and electrical conductivity of the hydrogel. The resulting material demonstrated exceptional self-healing ability, low hysteresis, and stretchability above 150% even after damage and repair. A key innovation lies in the post-treatment with NaCl, which induces a “salting-out” effect that strengthens intermolecular forces within the hydrogel. It promotes conformational changes in PEDOT chains from a benzoid to a more linear quinoid structure, increasing charge delocalization and hence electrical conductivity. The Raman spectroscopy analysis confirmed this conformational shift, and thermogravimetric analysis (TGA) revealed improved thermal stability of the composite hydrogels. From a mechanical standpoint, the tensile strength and elongation at break were significantly influenced by PEDOT:PSS content. The hydrogel with 4 mL of PEDOT:PSS achieved the best electrical performance and minimal hysteresis, although it slightly compromised stretchability. A trade-off was clearly observed between mechanical flexibility and conductivity: higher PEDOT:PSS loading improved conductivity but reduced elongation capacity due to potential aggregation within the polymer matrix (Figure 11). Electrically, the optimized hydrogels maintained excellent performance under strain. For example, the hydrogel showed a very low resistance change (ΔR/R_0_) of only 20% at 100% strain. It also maintained a linear current-voltage response, indicating stability in conductive pathways during deformation. This low hysteresis performance is crucial for consistent sensor output, especially in dynamic environments such as wearable applications [55,56,57,58].

**Figure 12:**
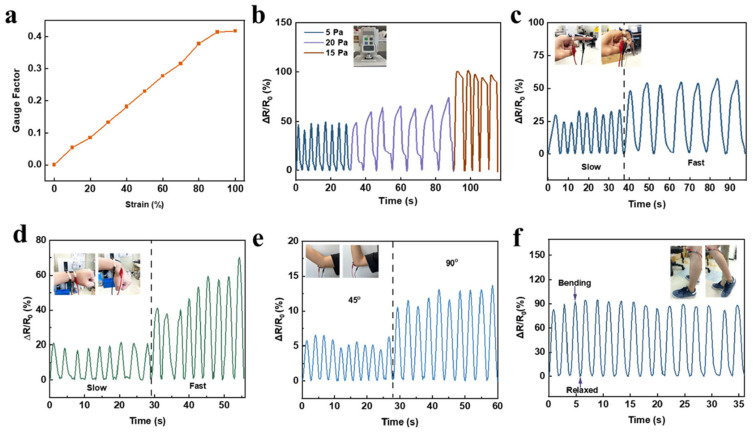
(**a**) Gauge factor of the PVA/PEDOT:PSS hydrogel under different strains. Sensing responses of the sensors for (**b**) pressure and (**c**) finger, (**d**) wrist, (**e**) elbow, and (**f**) knee bending [55].

The self-healing behavior was also rigorously evaluated. After mechanical damage, the hydrogel autonomously restored both its mechanical and electrical properties within 2 h at room temperature, without external stimulation. The reversible hydrogen bonding between the components was the primary mechanism of healing. Notably, the healed hydrogel could still achieve elongation at break exceeding 150%, with only minor decreases in tensile strength and modulus compared to the original state. Furthermore, sensor tests demonstrated practical viability. The hydrogel sensors were successfully used to detect various human motions, including wrist bending, knee movement, and even subtle facial expressions, as shown in Figure 12. Real-time monitoring showed response times as fast as 0.88 s, along with energy-efficient operation below 180 μW. These features make the material highly suitable for next-generation health monitoring, motion tracking, and human–machine interfaces [59,60]. The humidity sensitivity of the hydrogel also emerged as a bonus functional trait. The sensor responded reliably to moisture during respiration monitoring. The PSS component, being hygroscopic, absorbs water molecules from exhaled air, which in turn alters the resistance of the hydrogel. This property opens avenues for its use in breath analyzers and environmental humidity sensing.

In conclusion, the study makes a substantial contribution to the field of stretchable electronics. The combination of NaCl post-treatment, a smart polymer matrix, and an engineered freeze–thaw cross-linking method resulted in a hydrogel that balances stretchability, electrical performance, and self-healing—three characteristics that are often difficult to achieve together. The research opens up scalable opportunities for wearable sensors that are not only flexible and responsive but also durable and energy efficient. In summary, Prameswati et al. successfully engineered a high-performance, self-healing, and conductive hydrogel with exceptional stretchability and ultralow hysteresis. Its superior mechanical resilience, reliable strain sensing capabilities, and biocompatibility position it as a next-generation material for wearable electronics, soft robotics, and health-monitoring devices. The hydrogel’s combination of stretchability, sensitivity, self-repair, and long-term durability addresses key limitations of existing hydrogel-based sensors and paves the way for practical, sustainable, and intelligent soft electronic systems.

### 1.7. Stretchable Biodegradable Dual Cross-Linked Chitin Hydrogels with High Strength and Toughness and Their Potential Applications

As demand grows for sustainable and mechanically robust materials in biomedical engineering and flexible electronics, Wang et al. (2023) [61] developed an innovative hydrogel that achieves a rare combination of high strength, excellent stretchability, and complete biodegradability. The study introduces a novel dual cross-linked hydrogel based on carboxyethyl chitin (CECT) and polyacrylamide (PAAm), engineered to overcome the limitations of traditional hydrogels that often suffer from poor mechanical performance or environmental incompatibility. By integrating covalent and ionic cross-linking mechanisms, the researchers created a material that is not only tough and elastic but also biocompatible and environmentally friendly, making it an ideal candidate for emerging wearable electronics, wound dressings, and tissue scaffolds.

**Figure 13:**
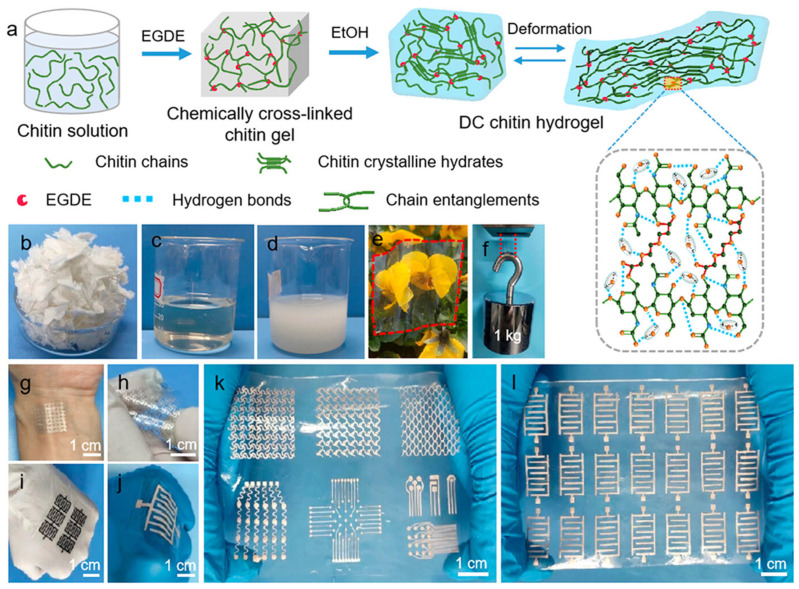
(**a**) Schematic of DC chitin hydrogel synthesis via sequential chemical and physical cross-linking. Photographs show: (**b**) purified shrimp shell patches, (**c**) chitin solution, (**d**) chitin-EGDE precursor, (**e**) transparent dual cross-linked hydrogels, and (**f**) a DC chitin strip (10 mm wide, 0.3 mm thick) supporting a 1 kg weight. Additional images include: (**g**) skin-attached hydrogel-printed electronics, (**h**) stretchable electronics, (**i**,**j**) supercapacitors printed with graphene and silver inks on a gloved fist, and (**k**,**l**) large-area, mass-produced hydrogel-printed electronics [61].

The hydrogel was fabricated using a combination of covalent and ionic cross-linking strategies, forming a highly resilient interpenetrating polymer network (IPN). The primary polymer, carboxyethyl chitin (CECT), was derived from natural chitin—a biodegradable biopolymer found in crustacean shells—functionalized to improve its solubility and reactivity [61,62,63,64]. This modified chitin was blended with acrylamide monomers and cross-linked with N,N′-methylenebisacrylamide (MBAA) to form the covalent network, while calcium ions (Ca^2^⁺) introduced dynamic ionic interactions by coordinating with the carboxylate groups on CECT. This dual-cross-linked architecture endowed the hydrogel with exceptional stretchability (up to 1400% elongation at break) and high tensile strength (up to 1.01 MPa), far exceeding typical performance metrics of biodegradable hydrogels, as shown in Figure 13a–l. The two networks played complementary roles: the covalent bonds provided permanent structural support, while the calcium-mediated ionic cross-links acted as reversible sacrificial bonds that dissipated energy during deformation. This dual-cross-linking approach mimicked the load-bearing strategies seen in biological tissues such as cartilage and skin, allowing the hydrogel to withstand high strain without mechanical failure. SEM imaging confirmed a homogenous, dense microstructure that further contributed to the enhanced mechanical properties [63,64,65].

**Figure 14:**
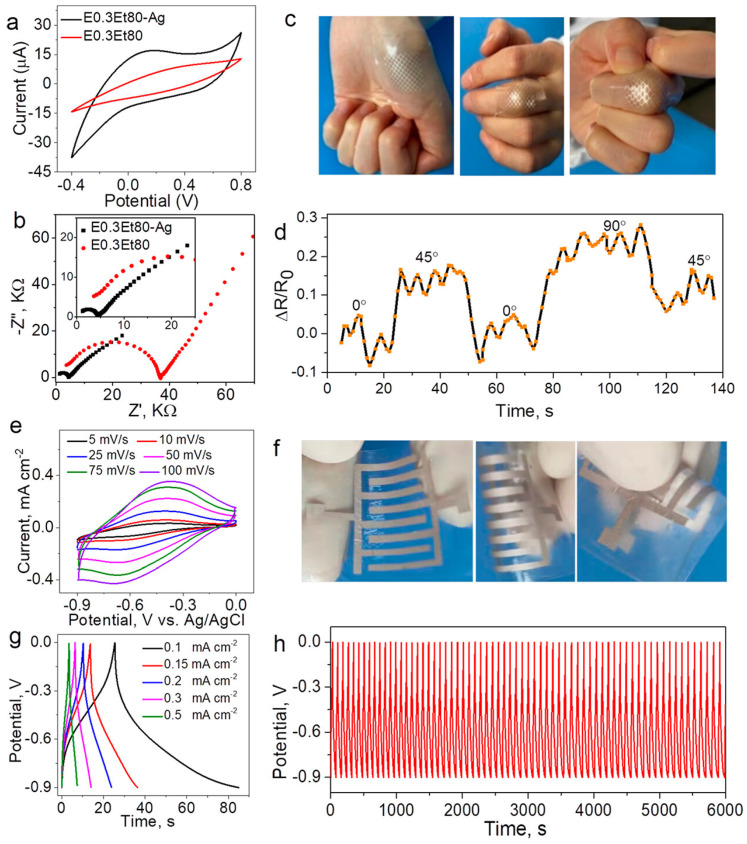
(**a**) CV curves and (**b**) Nyquist plot for electrochemical analysis using E0.3Et80 hydrogel and screen-printed E0.3Et80-Ag electrodes in deionized water. (**c**) Photo of dual cross-linked chitin hydrogel electronics on skin and (**d**) resistance variation with finger bending angles. (**e**) CV curves of the silver supercapacitor at various scan rates in 1 M Na_2_SO_4_. (**f**) Image of a foldable, flexible silver supercapacitor using screen-printed E0.3Et80 hydrogel. (**g**) GCD curves at different current densities and (**h**) cycling performance at 0.1 mA cm^−2^ [61].

One of the defining features of the hydrogel was its remarkable mechanical toughness and ability to recover from deformation. Tensile and compression tests showed that the material not only had a high breaking point but also excellent resilience, making it suitable for dynamic mechanical applications. When stretched or compressed, the hydrogel maintained its integrity over multiple loading-unloading cycles, indicating low hysteresis and long-term stability, as shown in Figure 14a–h. The ionic cross-links contributed to energy dissipation, absorbing mechanical energy through reversible bond breakage. Upon stress removal, these bonds reformed, allowing the hydrogel to recover its original structure. This energy dissipation behavior was particularly important for applications such as strain sensors, wearable devices, or tissue scaffolds that must maintain performance under repetitive mechanical loading. Rheological studies also demonstrated high elasticity and a wide linear viscoelastic range, meaning the hydrogel could deform significantly while maintaining mechanical coherence [64,65,66].

In contrast to synthetic hydrogels made from petroleum-based polymers, the CECT/PAAm hydrogel is fully biodegradable. Degradation studies using lysozyme solution revealed that the hydrogel could be broken down under physiological conditions, with the degradation rate controllable by tuning the CECT content or the cross-linking density. This feature makes it highly suitable for temporary biomedical implants or drug delivery systems that require materials to naturally degrade within the body after fulfilling their function.

**Figure 15:**
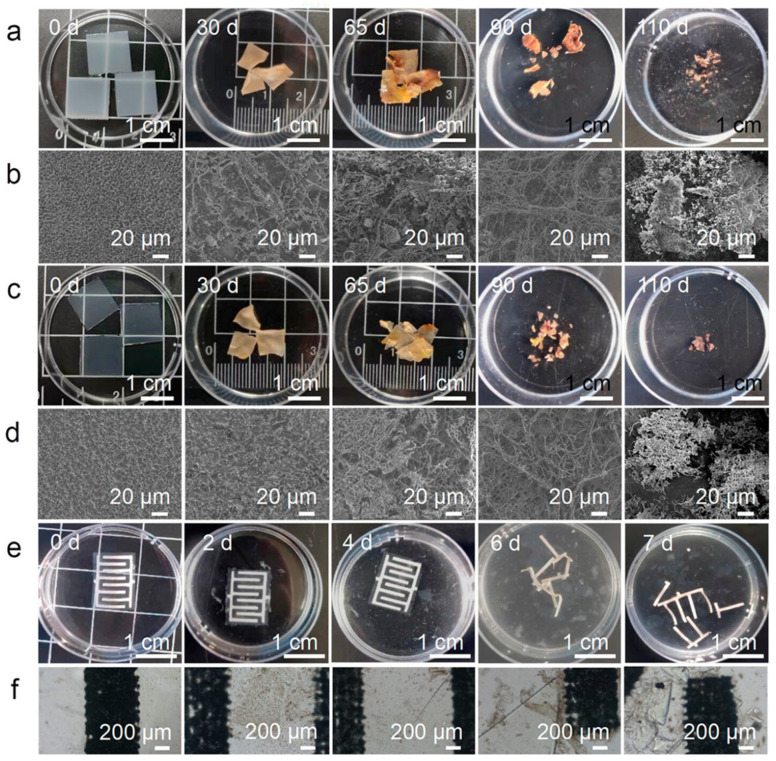
Soil degradation of chitin hydrogels and flexible electronics. Digital and FE-SEM images show (**a**,**b**) E0Et80 and (**c**,**d**) E0.3Et80 chitin hydrogels before and after soil degradation over 30, 65, 90, and 110 days. (**e**,**f**) Photographs and light microscopy images depict the biodegradation and enzymatic separation of E0.3Et80-Ag flexible electronics using 0.2 UN/mL chitinase solution [61].

Furthermore, the hydrogel exhibited excellent biocompatibility (Figure 15a–f) as evidenced by in vitro cell viability tests using NIH 3T3 fibroblast cells. The cells adhered well and proliferated on the hydrogel surface, suggesting that the material would not provoke cytotoxic effects or immune responses when applied to biological tissues. This biocompatibility, along with its water-rich, soft texture, enhances its potential for use in skin-contact applications such as wound healing patches, artificial cartilage, or implantable sensors. To demonstrate real-world applicability, Wang et al. integrated the hydrogel into a flexible, skin-mounted strain sensor. When attached to the human body, such as the knuckle, wrist, or elbow, the sensor was able to detect and respond to various human motions with high precision. The hydrogel’s electrical resistance changed linearly with strain, enabling accurate monitoring of bending and stretching. Its fast response time, low hysteresis, and high sensitivity made it suitable for capturing subtle human motions such as finger bending or facial expressions [61,62,63].

Importantly, the hydrogel maintained its sensing performance under repeated mechanical cycling, and its softness and adhesiveness provided comfortable skin contact. Unlike rigid sensors, the stretchable hydrogel conformed well to complex body surfaces, enhancing the user experience. Its self-recovery behavior also extended the device’s operational lifespan, which is crucial for long-term wearable electronics.

The material’s development reflects a broader trend toward sustainability in materials science. By leveraging a naturally abundant resource—chitin—and avoiding harmful solvents in the synthesis process, Wang et al. created a green alternative to conventional hydrogels. The fabrication method is scalable, low-cost, and environmentally benign, aligning well with future goals for biodegradable and eco-friendly medical and wearable devices. The hydrogel also shows potential for integration into soft robotics, smart clothing, and bioresorbable electronics. Wang et al.’s study represents a significant advancement in the development of stretchable, biodegradable hydrogels with multifunctional capabilities. By employing a dual cross-linked strategy, the researchers successfully engineered a hydrogel that bridges the gap between mechanical robustness and environmental compatibility. Its superior stretchability, toughness, and responsiveness position it as a promising candidate for flexible electronics, biomedical devices, and sustainable engineering materials. Looking ahead, this work lays a strong foundation for further innovations in hydrogel design. Future studies may focus on incorporating bioactive molecules, enhancing self-healing capabilities, or integrating the hydrogel into more complex bioelectronic systems. Regardless of the direction, this biodegradable, high-performance hydrogel represents a key step forward in the development of next-generation smart materials.

### 1.8. Ultradurable Noncovalent Cross-Linked Hydrogels with Low Hysteresis and Robust Elasticity for Flexible Sensors

**Figure 16:**
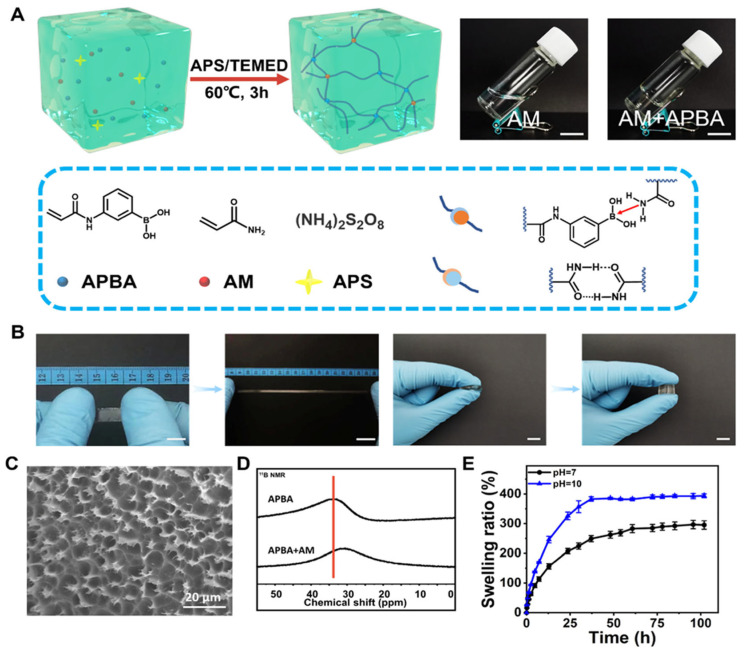
(**A**) Preparation scheme of P(AM_x_–APBA_y_) hydrogels and photos of PAM2 and P(AM2–APBA0.06) in water (scale bar: 1 cm). (**B**) Images of P(AM3–APBA0.06) hydrogel under stretching and compression (scale bar: 1 cm). (**C**) SEM image of P(AM2–APBA0.06). (**D**) ^11^B NMR spectra of APBA and its mixture with AM (DMSO-d_6_/D_2_O = 1:2). (**E**) Swelling behavior of P(AM3–APBA0.06) in buffers at pH 7 and 10 [67].

As the demand for high-performance, skin-compatible materials continues to rise in the field of flexible electronics, Xu et al. (2022) [67] present a significant advancement in the field of soft materials through the development of ultradurable noncovalent cross-linked hydrogels designed for flexible electronics. The study introduces a hydrogel system based on B–N coordination, which functions as the primary noncovalent cross-linking mechanism between acrylamide (AM) and 3-acrylamidophenylboronic acid (APBA). The resulting hydrogels, denoted as P(AMx–APBAy), were synthesized via a one-pot free radical polymerization method and showed exceptional mechanical performance, as shown in Figure 16. Notably, the B–N coordination exhibited strong stability in aqueous solutions, enabling the hydrogel to retain more than 95% of its tensile stress over 500 cycles of 200% strain, while maintaining a hysteresis below 10%. These features point to outstanding elasticity and mechanical resilience, making the hydrogel ideal for applications in wearable sensors and electronic skin. A key innovation is the incorporation of NaCl into the hydrogel network, which significantly enhances both the mechanical properties and the electrical conductivity of the material. With the addition of 1.72 M NaCl, the hydrogel reached a tensile strength of 0.21 MPa, a fracture strain of 1600%, and an electrical conductivity of 4.8 S/m—surpassing many previously reported ionic hydrogels. Furthermore, this modified hydrogel showed a high gauge factor of 10.2, indicating excellent sensitivity for strain sensing across both low (2.5%) and high (200%) deformation ranges. The integration of NaCl also improved the anti-freezing capability of the hydrogel, which remained flexible and functional at temperatures as low as −20 °C, making it suitable for harsh environmental conditions. Structurally, the SEM imaging in Figure 16C revealed a homogeneous porous network, which contributes to the hydrogel’s high stretchability and compressibility [67,68,69,70]. Rheological studies confirmed that the mechanical integrity remained stable across varying frequencies and temperatures, highlighting its thermal robustness. The hydrogel also demonstrated remarkable fatigue resistance during cyclic mechanical testing; both tensile and compressive tests showed negligible degradation in performance after 1000 cycles. This combination of low energy dissipation and high recoverability differentiates it from conventional hydrogen-bond-based hydrogels, which typically suffer from poor elasticity and rapid fatigue.

**Figure 17:**
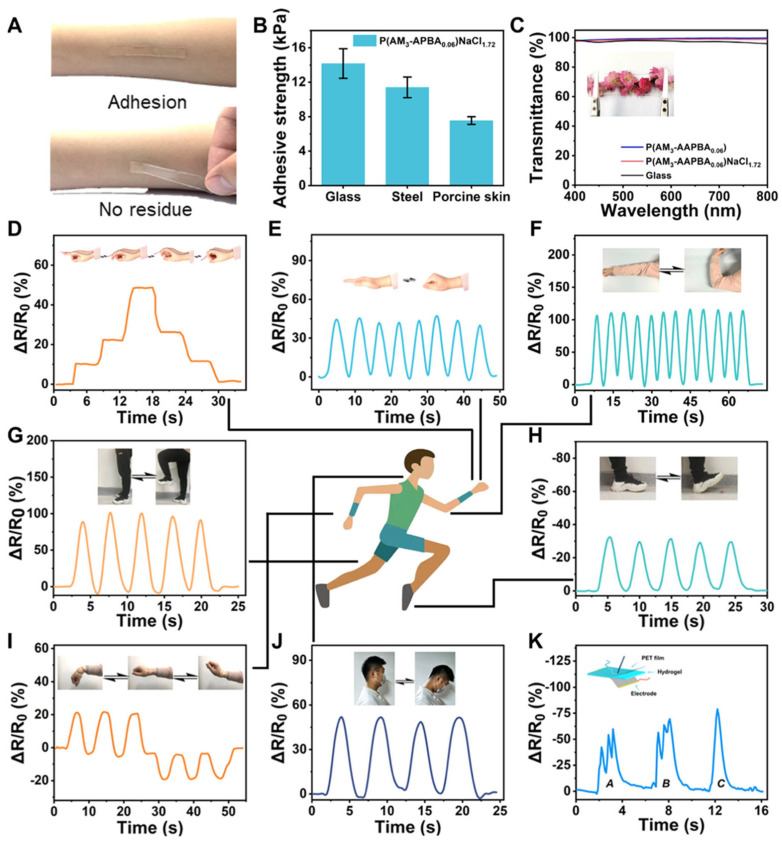
(**A**) The P(AM3–APBA0.06)NaCl1.72 hydrogel adheres well to skin without leaving residue. (**B**) Adhesion strength to glass, steel, and porcine skin. (**C**) Optical transmittance (400–800 nm) compared to glass. Relative resistance changes under various motions: (**D**) finger bending (0–90°), (**E**) fist clenching, (**F**) elbow bending, (**G**) knee movement, (**H**) foot motion, (**I**) wrist bending, and (**J**) neck bending. (**K**) Resistance changes during the handwriting of letters “A”, “B”, and “C” [67].

Another important feature of this hydrogel is its strong adhesion to biological tissues and a variety of substrates, including glass and metal, without requiring external adhesives. This property enables practical integration onto the human body for real-time activity monitoring. In demonstration applications, the hydrogel-based sensors effectively captured complex motions such as finger bending, elbow movement, neck tilting, and even writing (Figure 17). These demonstrations confirmed the material’s ability to produce consistent and reliable electrical signals with low latency and minimal noise. Beyond its electrical and mechanical properties, the hydrogel also proved to be highly transparent, with over 95% light transmittance, supporting its potential use in visually unobtrusive devices. Importantly, in vivo biocompatibility assessments using rat models showed only mild inflammatory responses during the initial implantation phase, which gradually subsided, indicating that the hydrogel does not pose long-term biological risks. Histological analysis showed tissue recovery after 30 days, suggesting its safety for prolonged contact with biological systems.

The authors conclude that the synergy between B–N coordination and ion conductivity in this hydrogel formulation addresses key limitations found in previous noncovalent hydrogels, especially regarding hysteresis and mechanical degradation. Unlike hydrogen bonds or metal coordination, the B–N interaction provides stronger and more reversible cross-links, granting the hydrogel excellent resilience under repetitive strain [67,68,69,70,71,72]. The successful combination of mechanical toughness, flexibility, stretchability, conductivity, and biocompatibility underlines the hydrogel’s potential for next-generation wearable and implantable electronics.

Overall, this work represents a comprehensive and scalable approach to designing high-performance hydrogels and opens new avenues for their deployment in fields such as soft robotics, biomedical devices, and interactive human–machine interfaces. The study’s meticulous characterization—from chemical composition to mechanical performance and biological response—ensures a thorough understanding of the material’s capabilities and limitations. Xu et al.’s work not only contributes a novel hydrogel formulation but also sets a framework for future research exploring alternative dynamic coordination chemistries in soft matter systems. With further integration into electronic platforms and additional testing under real-world conditions, this hydrogel system could play a crucial role in the development of intelligent, flexible sensing technologies.

### 1.9. Easy-to-Prepare Flexible Multifunctional Sensors Assembled with Anti-Swelling Hydrogels

**Figure 18:**
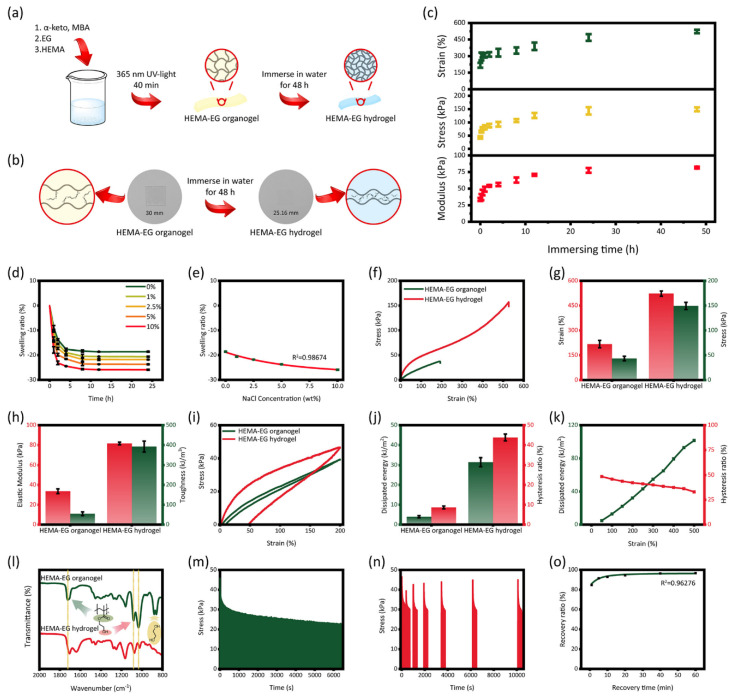
(**a**,**b**) Preparation and solvent exchange of HEMA-EG organogels to hydrogels. (**c**–**e**) Mechanical changes and swelling in NaCl solutions. (**f**–**h**) Tensile, fracture, and toughness properties. (**i**–**k**) Cyclic performance and hysteresis. (**l**) FT-IR spectra. (**m**–**o**) Stretching durability and recovery behavior [73].

In the evolving landscape of wearable electronics and smart sensing systems, the stability and durability of flexible sensors are critical for real-world applications. However, many hydrogel-based sensors suffer from limitations such as swelling, dehydration, and mechanical degradation, particularly under wet or extreme environmental conditions. To address these persistent issues, Yang et al. (2023) [73] present an innovative approach to the fabrication of flexible electronic devices using specially engineered hydrogels. The authors address two long-standing challenges in the field of hydrogel-based electronics—namely, the issue of swelling in aqueous environments and the difficulty of integrating multiple sensing functions within a single device. Their solution lies in the development of a hydrogel composed of poly(2-hydroxyethyl methacrylate) (poly-HEMA) prepared through a solvent replacement strategy. This hydrogel, referred to as HEMA-EG, shows not only remarkable swelling resistance but also enhanced mechanical performance and strong self-bonding capabilities, making it ideally suited for wearable and underwater sensor applications, as shown in Figure 18 [73,74,75].

The synthesis of the HEMA-EG hydrogel begins with the polymerization of HEMA in ethylene glycol, followed by immersion in water to induce solvent replacement. This process leads to the formation of new hydrogen bonding and hydrophobic interactions within the polymer network, resulting in a material with excellent dimensional stability and toughness. Unlike conventional hydrogels that expand significantly in aqueous environments, the HEMA-EG hydrogel shrinks upon immersion in water and saline solutions. This behavior is attributed to the hydrophobic nature of poly-HEMA and the salting-out effect observed in sodium chloride solutions. As a result, the hydrogel maintains its integrity and structure even in harsh environments, a quality that is critical for applications in underwater electronics. Mechanical tests reveal that the hydrogel becomes significantly stronger after solvent replacement, with substantial increases in fracture stress, elongation at break, and toughness.

Furthermore, cyclic stretching experiments demonstrate that the hydrogel maintains its mechanical properties over 500 cycles of 50% strain, exhibiting excellent fatigue resistance and self-recovery. These properties are essential for long-term use in flexible devices, where repeated deformation is inevitable. The modulus of the hydrogel, measured at approximately 81.63 kPa, is within the range suitable for biological soft tissues, further enhancing its potential for biomedical applications. A notable characteristic of the HEMA-EG hydrogel is its ability to self-bond without the need for adhesives or complex processing. When two pieces of hydrogel are brought into contact, they adhere to each other strongly, and this bond remains stable even after immersion in water. This self-bonding ability stems from hydrogen bonding interactions and hydrophobic effects within the polymer network. Such a property simplifies the assembly of flexible electronic devices, allowing complex sensor structures to be constructed without specialized tools or bonding agents [73,74,75,76,77,78].

**Figure 19:**
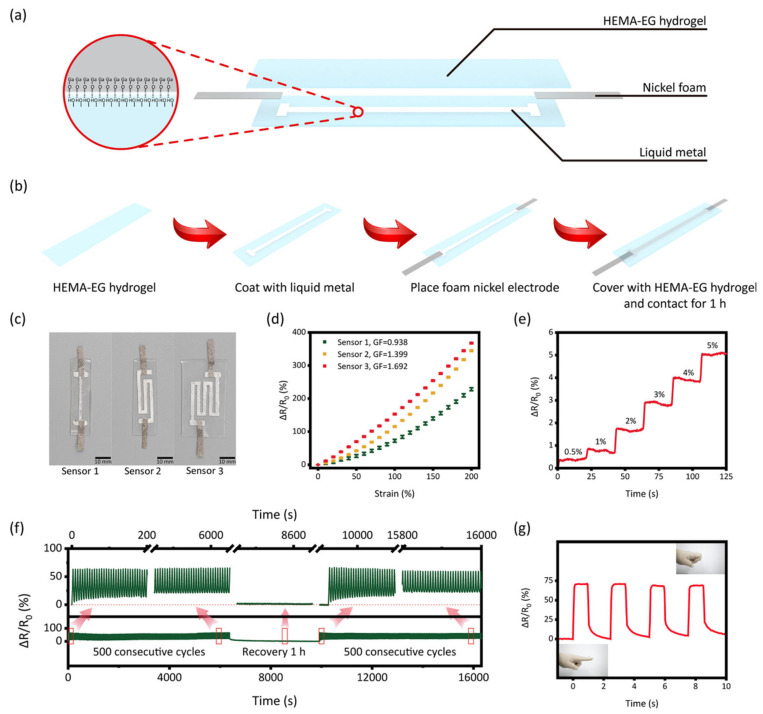
(**a**) Structure and (**b**) fabrication process of resistive strain sensors. (**c**) Images of three sensor samples. (**d**) Strain sensing performance. (**e**) Low-strain response of Sensor 3. (**f**) Cyclic stability of Sensor 3 at 50% strain. (**g**) Finger motion detection using Sensor [73].

Leveraging its self-bonding and mechanical properties, the HEMA-EG hydrogel was used to create both resistive and capacitive sensors. Resistive sensors were fabricated by applying liquid metal onto the hydrogel surface, with nickel foam serving as electrodes (Figure 19). These sensors demonstrated high sensitivity, were capable of detecting minimal strains as low as 0.5%, and maintained consistent performance across hundreds of stretch cycles. Among the various designs, a sensor with a more complex liquid metal pattern exhibited the highest sensitivity, highlighting the influence of structural configuration on performance. Capacitive sensors were also developed using the same self-bonding strategy to stack multiple hydrogel layers. These sensors showed stable and sensitive responses to strain and touch. The capacitive touch sensor, in particular, responded reliably to repeated physical contact, suggesting potential applications in electronic skin technologies. The versatility of the hydrogel in forming different types of sensors underscores its utility in multifunctional sensing systems.

To explore multidimensional sensing, the authors constructed two- and three-dimensional sensor arrays. A two-channel sensor was capable of determining the direction of strain based on changes in resistance, while a three-dimensional sensor with a 16-point grid could localize pressure points with high precision. These advanced sensing capabilities are made possible by the hydrogel’s robust self-bonding nature, which allows for simple but effective construction of intricate sensing networks. The culmination of this work is the development of a multifunctional underwater sensor. This device integrates strain sensing, temperature sensing, and water quality monitoring in a single multilayer structure. Its performance remains stable under water, demonstrating fast temperature response, sensitivity to ionic conductivity, and independence of function among the sensing modules [73,74,75,76,77,78]. Such integration is rarely achieved with hydrogel-based devices, especially in aquatic conditions. The success of this multifunctional sensor highlights the practical applicability of the HEMA-EG hydrogel in real-world scenarios, including environmental monitoring and underwater health tracking. In conclusion, this study introduces a high-performance, anti-swelling hydrogel with self-bonding properties that simplify the construction of flexible and multifunctional sensors. The HEMA-EG hydrogel overcomes critical limitations in hydrogel electronics and sets a precedent for designing robust, versatile, and easy-to-assemble devices for use in both terrestrial and underwater environments. This work opens promising avenues for future research and development in the fields of wearable electronics, soft robotics, and biomedical engineering.

Recent advancements in hydrogel systems reveal distinct strategies tailored to specific functional needs. Some hydrogels are designed for rapid responsiveness and self-healing, prioritizing dynamic adaptability for wearable electronics. Others emphasize mechanical toughness and durability through dual cross-linking techniques, aiming for long-term structural integrity under stress. This contrast underscores a common trade-off in hydrogel design: materials engineered for high flexibility often sacrifice mechanical strength, while those built for robustness may lack responsiveness. In biomedical applications, certain hydrogels are optimized for sustained drug release and biocompatibility, often using natural polymers to enhance degradation control and cell compatibility.

Meanwhile, other formulations focus on structural support in tissue engineering, incorporating scaffolding features that better mimic extracellular matrices. The functional objective—whether controlled therapeutic delivery or physical tissue support—directly influences the material composition and cross-linking strategy used. Energy harvesting applications present another comparison. Some hydrogels integrate ionic conductivity and moisture absorption for ambient power generation, prioritizing environmental responsiveness. Others, developed for electronic skin or biosensors, focus more on consistent signal transmission and sensitivity. Here, the design tension lies between maximizing energy conversion efficiency and ensuring mechanical stability under dynamic movement. When comparing adhesion strategies, hydrogels utilizing reversible bonding mechanisms exhibit strong skin adherence and reusability, while those with permanent cross-links provide more stable but less adaptable interfaces. Similarly, in terms of fabrication, simple one-step polymerizations offer scalability but may limit functional tunability compared to more intricate, multi-component synthesis processes. Ultimately, these varied approaches illustrate the balancing act between electrical performance, mechanical behavior, and fabrication complexity. A critical comparison of these works reveals not just incremental improvements, but divergent paths based on application-specific demands. Understanding these trade-offs is essential for guiding future research toward multifunctional hydrogels that effectively integrate performance, usability, and sustainability. Table 1 and Table 2 summarizes key properties of different hydrogels prepared.

## 2. Conclusions and Perspective

Recent developments in transparent hydrogel-based wearable electronics have shown promising strides toward unifying mechanical flexibility, electrical conductivity, optical transparency, and environmental compatibility within a single device architecture. Across the studies examined, performance metrics such as optical transmittance (>90%), electrical conductivity (~1.1 to 7.1 S/m), and stretchability (up to 1400%) stand out as critical KPIs for evaluating material readiness in wearable and biointegrated systems. For instance, silver nanowires, PEDOT:PSS, and MXene nanosheets demonstrated outstanding transmittance and conductivity, enabling their integration into visually imperceptible, skin-compatible electronics. Hydrogels such as those developed by Liu et al. (2024) [37], Prameswati et al. (2023) [55], and Wang et al. (2023) [61] exhibited superior toughness (>4800 J/m^2^), high elongation at break, and ultra-low hysteresis (~4.6%), signifying their suitability for use under continuous strain and motion. These mechanical KPIs are especially relevant for applications such as motion tracking, muscle sensing, or rehabilitation monitoring, where long-term conformability and fatigue resistance are crucial.

Moreover, the ability of some materials to maintain over 90% of their mechanical and electrical performance after hundreds to thousands of deformation cycles highlights their reliability for long-term use. Another essential KPI includes the hydrogel’s capacity for self-healing and repeatable adhesion—both were demonstrated to exceed 90% recovery rates in some systems. These characteristics ensure robustness in dynamic environments, reduce device replacement frequency, and support user comfort without additional adhesives. In terms of power generation, devices such as the Flexible Moisture-Electric Generator (FMEG) achieved an open-circuit voltage near 1 V under ambient conditions, offering a pathway toward battery-free, self-powered sensors for on-skin applications.

Despite these promising metrics, key challenges remain in achieving optimal balance among transparency, conductivity, and stretchability. Notably, materials with high optical transmittance often exhibit lower electrical conductivity or mechanical robustness compared to opaque or rigid counterparts. As a result, hybrid designs must refine percolation networks and bonding interfaces to overcome this trade-off. Fabrication challenges also persist: scalable, multi-layered transparent electronics require deposition methods that avoid damaging pre-existing layers while ensuring consistent layer alignment and electrical contact.

The need for novel transparent encapsulation materials with high stretchability and durability is particularly pressing in the context of soft robotics, augmented reality, and implantable medical devices. System integration offers further opportunity: AI-assisted multimodal sensors capable of capturing and distinguishing biosignals through a single, conformal device could significantly simplify hardware complexity while enhancing functionality. Looking ahead, materials that demonstrate not only high KPIs in isolated domains but also synergistic performance across electrical, mechanical, and optical benchmarks will drive the future of wearable electronics. Emphasis should also be placed on sustainability, where solvent-free fabrication, biodegradability, and low-energy processing methods can align hydrogel development with environmental goals. In conclusion, future innovations must focus on resolving the remaining material and integration limitations, enabling transparent, stretchable, and multifunctional electronics that seamlessly merge with the human body and the digital environment.

## Figures and Tables

**Table 1:** Key Features and Properties of the Various Hydrogels.

Hydrogel Type	Key Features	Mechanical Properties	Applications	Reference
Elastomeric ionic hydrogel	Moisture-electric generation, flexible, ionic conduction	Stretchable, durable under moist conditions	Wearable moisture-electric generators	Guchait et al. 2024 [25]
Bilayer Janus hydrogel	Janus properties, tunable modulus	High tensile strength, anisotropic behavior	Biosensors, soft robotics	He et al. 2022 [31]
Polyelectrolyte hydrogel	Piezoelectricity, adhesion, polyelectrolyte network	Good stretchability, piezoelectric response	Soft electronics, skin adhesion	Liu et al. 2024 [37]
MXene-based hydrogel	High toughness, puncture resistance, MXene reinforcement	Toughness ~1010 J/m^2^, puncture-resistant	Flexible electronics, wound protection	Lin et al. 2024 [43]
Photomediated conductive hydrogel	One-pot formation, electrical conductivity	Flexible and conductive	Flexible conductive networks	Nguyen et al. 2023 [49]
Self-healing conductive hydrogel	High stretchability, ultralow hysteresis, self-healing	Stretchability up to 2100%, ultralow hysteresis	Soft robotics, wearable health monitoring	Prameswati et al. 2023 [55]
Dual-cross-linked chitin hydrogel	High strength and toughness, biodegradable	Strength ~1.4 MPa, toughness ~1400 J/m^2^	Implants, bioelectronics	Wang et al. 2023 [61]
Noncovalent cross-linked hydrogel	Ultradurable, low hysteresis, elastic	Low hysteresis, elastic recovery	Flexible sensors, actuators	Xu et al. 2022 [67]
Protein-based hydrogel (casein)	Biocompatible, sensor applications, protein-derived	Varied depending on protein modification	Flexible sensors for health monitoring	Xu et al. 2024 [17]
Anti-swelling HEMA hydrogel	Anti-swelling, tissue-adaptable modulus, multifunctional sensors	Self-bonding, stable modulus under water	Underwater sensors, e-skin	Yang et al. 2023 [73]

**Table 2:** Key Properties of the Various Hydrogels.

References	Gauge Factor (GF)	Conductivity	Strain Limit	Adhesion Strength	Notable Features
Guchait et al., 2024 [25]	Not reported	~9.37 × 10^−3^ S/cm	~950%	Not quantified, strong moisture-induced adhesion claimed	Moisture-electric generation; ionic conductivity; wearable applications
Liu et al., 2024 [37]	GF ≈ 1.8–5.6	~0.95 S/m	Up to 1300%	Shear adhesion: ~7.1 kPa	Piezoelectric + adhesive polyelectrolyte hydrogel
Nguyen et al., 2023 [49]	GF = 2.4	~10^−1^ S/cm	~500%	Lap shear: ~9.2 kPa	One-pot photopolymerized conductive hydrogel
Prameswati et al., 2023 [55]	GF ≈ 3.5	~3.8 S/m	Up to 1000%	Lap shear strength: 10–12 kPa	Highly stretchable, self-healing, ultralow hysteresis
Xu et al., 2022 [67]	Not specified	Not highlighted	>1500%	Not reported	Ultralow hysteresis (<0.1 MJ/m^3^), superior elasticity
Xu et al., 2024 [17]	GF up to 6.8	~0.83 S/m	1350%	Adhesive to multiple substrates, >10 kPa	Protein-based sensors; tough, elastic, biocompatible
